# Prevalence and Determinants of Anorectal Disorders in Adult Patients with Urinary Incontinence: A Retrospective Cohort Study

**DOI:** 10.3390/jcm15031131

**Published:** 2026-02-01

**Authors:** Reem Alharbi, Ebtesam Almajed, Norah Alqntash, Ebtihag O. Alenzi, Rawan Bin Salamah, Reem Altamimi, Kayan Alotaibi

**Affiliations:** 1 Surgery Department, College of Medicine, Princess Nourah bint Abdulrahman University, Riyadh 11671, Saudi Arabia; 2 Department of Clinical Sciences, College of Medicine, Princess Nourah bint Abdulrahman University, Riyadh 11671, Saudi Arabia; 3 Family and Community Medicine Department, College of Medicine, Princess Nourah bint Abdulrahman University, P.O. Box 84428, Riyadh 11671, Saudi Arabia; eoalenzi@pnu.edu.sa

**Keywords:** urinary incontinence, anorectal dysfunction, pelvic floor disorders

## Abstract

**Background/Objectives:** Pelvic floor dysfunction (PFD) encompasses urinary incontinence (UI), anorectal disorders (ARDs), and other related conditions that frequently coexist. Evidence on the prevalence and determinants of ARD among patients with UI, particularly in Middle Eastern populations, remains limited. This study aimed to assess the prevalence of ARD in UI patients and identify key clinical and demographic factors associated with anorectal disorders. **Methods:** A retrospective cohort study was conducted between January 2017 and June 2025. Consecutive adult patients diagnosed with UI were included. Demographic, clinical, obstetric, and surgical characteristics were extracted from medical records. Categorical comparisons were performed using chi-square and Fisher’s exact tests, while predictors of ARD were analyzed using multivariable logistic regression. **Results:** Among 494 patients with UI, ARD was present in 115 cases, yielding a prevalence of 23.3%. The most frequent ARD diagnoses were hemorrhoids (29.8%), obstructive defecation syndrome (19.3%), and fecal incontinence (18.1%). Patients with ARD more often had stress UI (25.2% vs. 18.5%) and overflow UI (4.3% vs. 2.1%) compared with those without ARD. Chronic constipation was significantly associated with ARD (39.7% vs. 10.7%, *p* < 0.001), as were hypothyroidism (31.8% vs. 21.4%, *p* = 0.037), psychological conditions (37.1% vs. 21.0%, *p* = 0.003), and sexual dysfunction (64.7% vs. 19.0%, *p* < 0.001). Logistic regression identified rectocele (aOR = 6.54, 95% CI: 3.18–13.45, *p* < 0.001) and previous pelvic surgery (aOR = 2.27, 95% CI: 1.17–4.41, *p* = 0.016) as independently associated with ARD. Increasing age was inversely associated with ARD (aOR = 0.98, 95% CI: 0.96–0.99, *p* = 0.014). **Conclusions:** Our findings underscore the need to incorporate anorectal and sexual health screening in UI patients. Early recognition, coupled with multidisciplinary team-based management, may help optimize outcomes, enhance treatment compliance, improve quality of life, and reduce the long-term burden associated with PFD.

## 1. Introduction

Pelvic floor dysfunction (PFD) encompasses a spectrum of disorders resulting from impaired pelvic floor muscle and connective tissue support, including urinary incontinence (UI), pelvic organ prolapse, fecal incontinence, and pelvic pain [1,2]. These conditions are highly prevalent worldwide, with estimates suggesting that approximately 1.9% to 60% of women experience at least one pelvic floor disorder during their lifetime [1,3,4].

PFD imposes a substantial physical, psychosocial, and economic burden. Affected individuals often report embarrassment, reduced self-esteem, social withdrawal, and impaired quality of life due to limitations in daily activities, sexual dysfunction, and strained interpersonal relationships [1]. Psychological distress is also common; for example, UI has been significantly associated with depressive symptoms [1]. From a health system perspective, PFD contributes to increased healthcare utilization, including outpatient visits, medications, physiotherapy, and surgical interventions, resulting in considerable direct and indirect costs [5].

Anorectal disorders (ARDs) represent an important but frequently under-recognized component of PFD. Similarly to UI, ARDs increase with age and share common risk factors such as multiparity, obstetric trauma, and pelvic floor muscle weakness [6]. A recent systematic review and meta-analysis estimated the global prevalence of fecal incontinence to range between 6.8% and 9.2%, with higher rates among women and older adults [7]. However, data from the Middle East and Africa remain limited, underscoring a regional knowledge gap. Chronic functional constipation further illustrates the interrelationship between bowel and bladder dysfunction. Prior studies have demonstrated that women with functional constipation are significantly more likely to experience overactive bladder symptoms and urinary incontinence [8]. These findings support the concept that pelvic floor disorders often coexist and cluster clinically as a continuum involving both urinary and anorectal components. Despite this recognized overlap, most studies have traditionally examined urinary and anorectal disorders in isolation. Evidence remains scarce regarding the prevalence of ARD specifically among patients presenting with UI and the factors associated with this coexistence, particularly in Middle Eastern populations. We hypothesized that anatomical abnormalities (such as rectocele), prior pelvic surgery, and chronic constipation would be associated with a higher prevalence of anorectal disorders among patients with urinary incontinence. Therefore, this study aimed to determine the prevalence of ARD in patients diagnosed with UI and to identify associated demographic and clinical factors.

## 2. Materials and Methods

### 2.1. Study Design and Population

This retrospective cohort study was conducted in the Department of Surgery at a University Hospital in Riyadh, Saudi Arabia, between 1 January 2017 and 4 June 2025. Consecutive sampling was employed, based on patient records, to include all women with a diagnosis of urinary incontinence during the study period.

For the purposes of this study, anorectal disorders (ARDs) were defined as a heterogeneous group of anorectal conditions documented in the medical records, including pelvic floor–related disorders such as fecal incontinence, obstructive defecation, and rectal prolapse, as well as commonly coexisting anorectal conditions such as hemorrhoids and anal fissures. Although not all included conditions are exclusively classified as pelvic floor dysfunctions, they were considered relevant due to their frequent coexistence with urinary incontinence and their association with pelvic floor weakness, chronic constipation, or increased straining.

Patients were eligible if their medical records contained complete and accessible data. No restrictions were applied based on gender. Patients younger than 18 years and those without a diagnosis of urinary incontinence were excluded.

### 2.2. Ethics Statement and Informed Consent

The study was carried out in accordance with the ethical principles outlined in the Declaration of Helsinki. Ethical approval was obtained from the Institutional Review Board (IRB) of King Abdullah bin Abdulaziz University Hospital (IRB Log Number: 24-0017). Given the retrospective design of the study, which involved the review of existing medical records of patients diagnosed with UI, the requirement for informed consent was waived. All patients’ data were anonymized and handled in compliance with data protection and confidentiality regulations.

### 2.3. Data Extraction

The extracted data were categorized into four main domains: (1) Biometric and demographic information, which included patients’ current age (years), age at presentation (years), weight at presentation (kg), height at presentation (cm), and calculated body mass index (BMI, kg/m^2^). Demographic variables included gender, marital status, and smoking status. In addition, the presence of comorbidities, including diabetes mellitus (DM), hypertension (HTN), asthma, thyroid disorders (hyperthyroidism or hypothyroidism), dyslipidemia, cardiovascular diseases, neurological disorders (e.g., multiple sclerosis, Parkinson’s disease, peripheral neuropathy, and spinal cord injuries), and mental or psychological illnesses. (2) Clinical-related information, including the presence or absence of anorectal dysfunction, was extracted for each patient. Diagnoses were identified from clinical notes and confirmed by a colorectal consultant assessment; when anorectal disorder was present, the specific condition was documented. Similarly, the type of urinary incontinence (stress, urge, mixed, or overflow) was determined based on the urology consultant documentation, and the presence of pelvic organ prolapse (cystocele and rectocele) was recorded. (3) Obstetric history, including parity, number of cesarean sections, number of vaginal deliveries, weight of the largest infant, number of abortions, and the occurrence of anal sphincter tear (third- or fourth-degree perineal laceration). (4) Gynecological and anorectal surgical history, which includes menstrual status and prior gynecological surgeries, including hysterectomy and prolapse repair whether vaginal or pelvic floor repair in general, was recorded. Previous anorectal surgeries, such as hemorrhoidectomy, were documented separately. Anorectal disorders were identified based on documentation in medical records following symptom-driven referral and evaluation by a colorectal consultant; routine systematic anorectal screening was not performed for all patients with urinary incontinence. Sexual dysfunction was identified retrospectively based on documentation in clinical notes when patients reported symptoms such as dyspareunia, decreased sexual desire, avoidance of sexual activity, or other sexual complaints during routine consultations; no standardized or validated sexual function questionnaires were used.

### 2.4. Outcome Measures

The primary outcome of this study was the presence of any anorectal disorder (ARD) among patients diagnosed with urinary incontinence. Secondary outcomes included the prevalence and distribution of specific anorectal disorder subtypes, as well as the association of ARD with selected demographic, clinical, obstetric, surgical, and pelvic floor-related variables, including sexual dysfunction and relevant comorbidities.

### 2.5. Statistical Analysis and Data Management

All data were coded, entered, and analyzed using the Statistical Package for the Social Sciences (SPSS), Version 25.0 (SPSS Inc., Chicago, IL, USA). Continuous variables were summarized as means with standard deviations (mean ± SD). Categorical variables were presented as frequencies and percentages. Comparisons between patients with and without anorectal disorder were conducted using Pearson’s chi-square test or Fisher’s exact test, as appropriate, for categorical variables. To further identify predictors of ARD, a multivariable logistic regression analysis was performed. The dependent variable was the presence of any anorectal dysfunction, while independent variables included sociodemographic characteristics, urinary incontinence subtypes, pelvic organ prolapse, and obstetric or surgical history. Adjusted odds ratios (aORs) with 95% confidence intervals (CIs) were calculated. A two-tailed *p*-value of <0.05 was considered statistically significant. Missing data were handled using complete-case analysis; observations with missing or “unknown” values were excluded from the logistic regression, and no imputation or separate “unknown” categories were created. Variables were selected for multivariable modeling based on clinical relevance and prior literature rather than solely on univariate *p*-values. Model assumptions were assessed before finalization: multicollinearity was evaluated and found to be acceptable, and linearity in the logit for continuous variables was examined and met. Overall model fit was confirmed using the Hosmer–Lemeshow test (*p* = 0.203).

## 3. Results

The study included 494 adult patients with urinary incontinence (UI). The main characteristics of our sample are shown in Table 1. The mean age was 53.8 ± 14.7 years, with most of the participants aged 50 years and older (62.2%). Women made up most of the sample (91.5%). Most participants were married (89.9%), with only a small percentage being non-married (10.1%). Regarding smoking status, most were non-smokers (95.7%), while former smokers represented the smallest group (1.2%).

The mean BMI was 30.9 ± 6.6 kg/m^2^; over half of the participants were classified as obese (55.5%), and the lowest proportion was in the underweight/normal weight category (17.8%). Regarding comorbid conditions, chronic constipation was most frequent (43.3%), followed by dyslipidemia (38.9%), diabetes (33.8%), and hypertension (33.8%). Recurrent urinary tract infections occurred in (23.5%). Hypothyroidism was reported by (17.8%), asthma by (14.4%), and psychological conditions by (14.2%); among them, depression (7.6%) and anxiety (7.7%) were the most common (not tabulated). Less common were cardiovascular diseases (6.9%), neurological diseases (30, 6.1%), and hyperthyroidism (1.0%).

**Figure 1:**
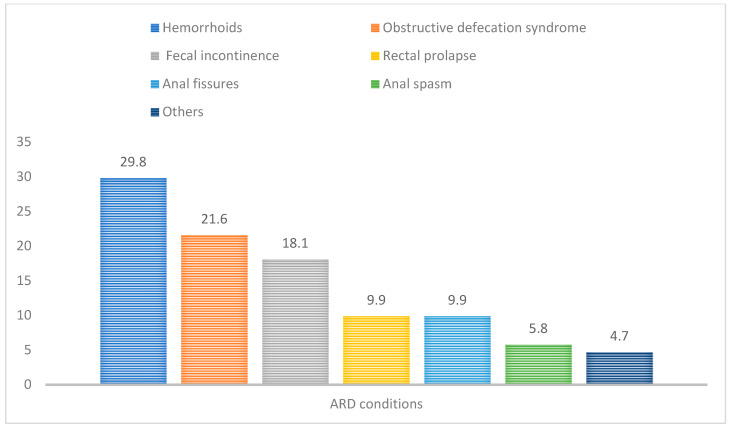
Most Commonly Reported Types of Anorectal Disorder Among Patients with Urinary Incontinence.

Among the 494 patients with urinary incontinence, 115 patients were diagnosed with at least one anorectal disorder, yielding an overall ARD prevalence of 23.3% (Table 2). Within the ARD subgroup (*N* = 115), most patients had a single anorectal condition (64.3%), while 21.7% had two conditions, 11.3% had three conditions, and 2.6% had four or more coexisting anorectal conditions. When examining the distribution of specific anorectal disorders within the ARD subgroup, the most frequently identified conditions were hemorrhoids (29.8%), obstructive defecation syndrome (21.6%), and fecal incontinence (18.1%). Rectal prolapse and anal fissures were each observed in 9.9%, followed by anal spasm (5.8%) and anal fistula (1.8%). The distribution of anorectal disorder types among patients with urinary incontinence is illustrated in Figure 1.

Clinical, obstetric, and pelvic floor characteristics of the study population are presented in Table S1. Urge UI was the most common type, followed by mixed, stress, and overflow. However, the distribution of UI types did not differ significantly between groups (*p* = 0.137). Compared with patients without ARD, those with ARD more often had stress UI (25.2% vs. 18.5%) and overflow UI (4.3% vs. 2.1%), less often had urge UI (33.9% vs. 44.6%), while mixed UI was similar (36.5% vs. 34.8%). In terms of pelvic organ prolapse, higher prolapse grades were more frequent among women with ARD. For cystocele, grade 1 was observed in 20.4% of women with ARD compared to 9.0% without ARD; grade 2 in 13.0% vs. 6.7%; and grade 3 in 9.3% vs. 4.9%. A similar pattern was seen for rectocele: grade 1 was present in 16.7% vs. 4.7%, grade 2 in 13.9% vs. 4.1%, and grade 3 in 10.2% vs. 1.2%, among those with and without ARD, respectively (*p* < 0.001).

Premenopausal status (60.2% vs. 45.9%; *p* = 0.011) and parity (84.3% vs. 75.3%; *p* = 0.014) were significantly more frequent among patients with anorectal disorders. Although the mean total number of deliveries was higher in the ARD group (4.19 ± 3.03 vs. 3.68 ± 3.30), this difference did not reach statistical significance (*p* = 0.153). Women with ARD also more frequently had three to four vaginal deliveries (30.6% vs. 20.1%), although the overall distribution of vaginal deliveries was not significantly different between groups (*p* = 0.100). Likewise, the mean largest infant birth weight was higher in women with ARD (3535.0 ± 576.9 g vs. 3360.7 ± 427.2 g), but this difference was not statistically significant (*p* = 0.210). Abortions were significantly more frequent among women with ARD (*p* < 0.001), and previous pelvic surgeries were also significantly more common in the ARD group (26.9% vs. 6.4%; *p* < 0.001). Anal sphincter tears were observed only in the ARD group (3.7% vs. 0.0%; *p* = 0.001), and sexual dysfunction was significantly more frequent among patients with ARD compared with those without ARD (18.5% vs. 2.3%; *p* < 0.001).

Differences between patients with and without anorectal disorders (ARDs) among adults diagnosed with urinary incontinence are presented in Table 3. Several variables showed statistically significant associations with the presence of ARD, including age group (*p* = 0.007), hypertension (*p* = 0.014), hypothyroidism (*p* = 0.037), mental or psychological conditions (*p* = 0.003), chronic constipation (*p* < 0.001), previous pelvic surgery (*p* = 0.003), anal sphincter tear (*p* = 0.005), and sexual dysfunction (*p* < 0.001). When stratified by age group, the highest proportion of ARD was observed among participants aged 40 to <50 years (33.0%), while the lowest proportion was seen among those aged ≥65 years (13.3%) (*p* = 0.007). Regarding hypertension, ARD was more frequent among participants without hypertension (26.6%) compared with those with hypertension (16.8%) (*p* = 0.014). Participants with hypothyroidism had a higher prevalence of ARD (31.8%) than those without hypothyroidism (21.4%) (*p* = 0.037).

A significantly higher proportion of ARD was observed among participants with a mental or psychological condition (37.1%) compared with those without (21.0%) (*p* = 0.003). Chronic constipation showed the strongest association with ARD, with 39.7% of participants with constipation affected compared with 10.7% of those without constipation (*p* < 0.001). ARD was also more frequent among participants with a history of pelvic surgery (32.8%) compared with those without prior pelvic surgery (16.8%) (*p* = 0.003). Finally, sexual dysfunction was strongly associated with ARD, with 64.7% of participants reporting sexual dysfunction affected by ARD compared with 19.0% of those without sexual dysfunction (*p* < 0.001).

Table 4 presents the results of the multivariable logistic regression analysis examining predictors of ARD in the 494 UI patients. Among sociodemographic variables, age was the only significant factor; increasing age was associated with a slight reduction in the odds of ARD (aOR = 0.977, 95% CI: 0.959–0.995, *p* = 0.014). Neither BMI, gender, nor marital status showed statistically significant associations.

Regarding urinary incontinence subtypes, none were independently predictive of ARD in the adjusted model. Overflow incontinence had the highest odds ratio (aOR = 2.143, 95% CI: 0.393–11.689), but the association was not statistically significant (*p* = 0.379). Rectocele emerged as a strong and statistically significant factor associated with ARD (aOR = 6.540, 95% CI: 3.179–13.454, *p* < 0.001), whereas cystocele was not significantly associated with ARD (aOR = 0.963, 95% CI: 0.481–1.926, *p* = 0.915).

In terms of clinical and obstetric history, previous pelvic surgery was significantly associated with increased odds of ARD (aOR = 2.265, 95% CI: 1.165–4.406, *p*= 0.016). Variables such as the number of deliveries (aOR = 1.124, 95% CI: 0.815–1.550), abortions (aOR = 0.846, 95% CI: 0.532–1.345), menstrual status (aOR = 0.951, 95% CI: 0.203–4.450), and the largest infant weight (aOR = 1.001, 95% CI: 1.000–1.003) did not reach statistical significance.

## 4. Discussion

Pelvic floor dysfunction (PFD) frequently coexists and may act as independent risk factors for each other [9]. The overlap between Urinary incontinence (UI) and anorectal disorder (ARD) has been underrecognized with a scarcity of comprehensive studies examining ARD among patients with UI. Therefore, this retrospective cohort study was designed to address this gap by determining the prevalence of ARD among UI patients and identifying its key determinants. The prevalence of UI among women has been reported to range from 29% to 45% [10,11]. Fecal incontinence is less frequent but still significant, affecting about 8% of adults globally [7]. Moreover, epidemiologic data indicates that POP prevalence ranges from 3% to 50%, and the global burden remains high, with approximately 13 million new cases reported in 2019 alone [12].

According to our findings, 23.3% of patients with UI had coexisting ARD, underscoring that nearly one in four individuals with UI also suffer concurrent anorectal symptoms. Additionally, the strongest association was observed with chronic constipation; patients reporting long-standing constipation were significantly more likely to have ARD, highlighting a strong clinical association between bowel symptoms and pelvic floor dysfunction. Furthermore, the presence of a rectocele was strongly associated with ARD in our UI cohort. A history of previous pelvic surgery emerged as another determinant, suggesting a potential link between prior gynecologic or colorectal surgeries may predispose patients to combined bladder-bowel dysfunction. Finally, we observed that sexual dysfunction was more common in UI patients with ARD. This overlap indicates that pelvic floor disorders frequently coexist with broader functional complaints, including sexual health issues. These results support consideration of a holistic clinical approach to UI patients, who should be evaluated not just for urinary symptoms but for anorectal and sexual health as well.

Our findings align with and extend the growing body of literature on the interrelationship between pelvic floor disorders. Prior studies have reported that multiple pelvic floor dysfunctions often coexist. Biswas et al. found that among women with UI presenting to care, 35.5% also had FI and 41.2% had constipation [13]. Similarly, Saraçoğlu et al. found that among 200 women with UI, 7.5% had clinical fecal incontinence, 77% experienced some degree of involuntary gas leakage, and over 52% reported chronic constipation [14]. These data are consistent with the ARD prevalence of 23.3% of our UI patients. Constipation has been prospectively associated with developing lower urinary tract symptoms such as urinary urgency and hesitancy in middle-aged women. Alhababi et al. reported that women with chronic constipation had a significantly higher risk of later urinary urgency and difficulty voiding [15]. In terms of sexual dysfunction, our study adds to a growing recognition that pelvic floor disorders rarely exist in isolation. While sexual function is not traditionally examined alongside UI and FI in epidemiologic studies, there is evidence that incontinence and pelvic floor disorders adversely affect sexual health. Bezerra et al. conducted a systematic review and found that women with incontinence report worse sexual function scores across multiple domains [16]. Similarly, Koparal et al. reported that double incontinence is associated with impaired body self-image and reduced self-confidence that can significantly diminish sexual intimacy and satisfaction [17]. Our finding of sexual dysfunction being linked to ARD in UI patients underscores the complexity of coexisting pelvic floor conditions and highlights an area that warrants further exploration. Although sexual dysfunction demonstrated a strong association with anorectal disorders in this cohort, this finding should be interpreted cautiously. Sexual dysfunction was identified through retrospective chart review rather than standardized assessment tools and therefore represents a hypothesis-generating observation. Prospective studies employing validated sexual function instruments are warranted to further elucidate the relationship between urinary incontinence, anorectal disorders, and sexual health.

While FI is often studied in comparison with UI, the present study included only patients with a confirmed diagnosis of urinary incontinence. As such, direct comparisons between individuals with and without urinary incontinence were not possible. Instead, FI was examined as a clinically relevant anorectal outcome within this population, allowing assessment of its prevalence and associated determinants among patients already affected by urinary incontinence.

Several determinants have been implicated in the co-occurrence of UI and ARD. Shared etiological factors such as pelvic floor muscle damage, pudendal nerve injury, or connective tissue laxity can arise from childbirth, surgical interventions, or neurological diseases, predisposing patients to both urinary and fecal control problems [18,19]. Epidemiological studies consistently identify advanced age and parity, especially those with a history of multiple or traumatic vaginal deliveries, as risk factors for both UI and FI [6]. Neurological comorbidities, including stroke or peripheral neuropathy, have also been strongly linked to dual incontinence [18]. In older women, the presence of multiple chronic illnesses and mental health factors like depression is associated with markedly higher odds of developing double incontinence [20]. In men, indicators of general frailty such as dependence in activities of daily living and poor self-rated health appear to be significant predictors of combined urinary and fecal incontinence [20].

This study has several limitations that should be noted. First, the retrospective design relies on the accuracy and completeness of medical records. This may have resulted in underreporting of certain clinical factors, such as constipation severity, obstetric complications, and sexual dysfunction. Importantly, sexual dysfunction was assessed retrospectively based on documented patient-reported symptoms rather than standardized questionnaires, which may have led to underreporting and limits the interpretability of this finding. Second, since this is a single-center study conducted in a University Hospital in Riyadh, the findings may not apply to the broader Saudi population or other healthcare settings, especially primary care or community-based groups. Additionally, some variables contained missing or unknown categories which may have introduced bias and limited the precision of the results. Also, no a priori sample size calculation or formal power analysis was performed, as the study was retrospective. It included all eligible patients with urinary incontinence identified during the study period. Although the final sample size was relatively large, this approach may limit the ability to detect smaller effect sizes. Future prospective studies with predefined sample size calculations are warranted to confirm these findings. Despite these limitations, the study offers new insights into the prevalence and causes of ARD in patients with urinary incontinence within a Middle Eastern population, highlighting an important gap in evidence.

Although anorectal disorders are commonly reported to increase with age, the inverse association observed in this study should be interpreted cautiously. Anorectal disorders were identified through review of documented diagnoses in medical records rather than through prospective systematic screening. Older adults may underreport anorectal symptoms, normalize them as part of aging, or prioritize other comorbidities during clinical encounters, leading to potential underdiagnosis. Variations in symptom reporting and clinical recognition across age and menopausal groups may therefore have influenced detection. Survivor bias may also contribute, whereby individuals with more sever pelvic floor dysfunction are less likely to be presented in older age groups. These findings likely reflect cohort-specific characteristics rather than a contradiction of established clinical patterns. Future prospective studies using age categorization or non-linear modeling are warranted to better investigate the relationship between age and anorectal disorders in patients with UI.

The clinical implications of our findings highlight that healthcare providers caring for patients with urinary incontinence should routinely include screening for anorectal disorders. A considerable number of UI patients have unrecognized anorectal disorders, which can continue to impair their quality of life if not addressed. Our results provide practical guidance for a multidisciplinary assessment; identifying chronic constipation, rectocele, and a history of pelvic surgery as a potential warning sign should prompt a comprehensive pelvic floor evaluation, including assessment of sexual function. From a patient care standpoint, addressing coexisting ARD in UI patients can result in more complete management plans. A multidisciplinary team (MDT) that includes urology, gynecology, colorectal surgery, pelvic floor physical therapy, and psychology can improve patient outcomes by ensuring a coordinated approach that addresses co-occurring problems. Recent studies indicate that a team-based strategy leads to better patient-reported results, greater treatment adherence, and a higher quality of life in managing pelvic floor disorders [21].

## 5. Conclusions

In conclusion, anorectal disorders (ARDs) are a common comorbidity among patients with urinary incontinence (UI). Chronic constipation, rectocele, and a history of pelvic surgery were significantly associated with the presence of anorectal disorders, highlighting the clinical overlap between bladder and bowel dysfunction. The observed association with sexual dysfunction highlights the frequent coexistence of pelvic floor-related conditions. Clinicians should systematically screen UI patients for anorectal disorders and sexual dysfunction and manage them through a multidisciplinary team to optimize outcomes and quality of life.

## Figures and Tables

**Table 1:** Sociodemographic Characteristics of the Studied Sample (*N* = 494).

Variables		*N*	%
Age			
	Mean ± SD	53.8 ± 14.7	
	18–29 years	30	6.1
	30–39 years	51	10.3
	40–49 years	106	21.5
	50–64 years	187	37.9
	≥65 years	120	24.3
Gender			
	Male	42	8.5
	Female	452	91.5
Marital Status			
	Ever married †	444	89.9
	Non-married	50	10.1
Smoking Status			
	Non-smokers	473	95.7
	Former smokers	6	1.2
	Current Smokers	15	3.0
BMI (kg/m^2^)			
	Mean ± SD	30.9 ± 6.6	
	Underweight/normal weight	88	17.8
	Overweight	132	26.7
	Obese	274	55.5
Comorbidities *			
	Diabetes	167	33.8
	Hypertension	167	33.8
	Asthma	71	14.4
	Hyperthyroidism	5	1.0
	Hypothyroidism	88	17.8
	Dyslipidemia	192	38.9
	Cardiovascular Disease (CVDs)	34	6.9
	Neurological Disease	30	6.1
	Psychological Condition	70	14.2
	Chronic Constipation	214	43.3
	Recurrent Urinary Tract Infections	116	23.5

* Participants could report more than one comorbidity; therefore, totals exceed *N*. † Ever married includes currently married, divorced, or widowed individuals.

**Table 2:** Prevalence and Burden of Anorectal Disorder Among Patients with Urinary Incontinence (*N* = 494).

Category		Frequency	Percentage
Presence of Any Anorectal Dysfunction		115	23.3
	Yes	74	64.3
	No	379	76.7
Coexisting Anorectal Disorders (*N* = 115)			
	Patients with one anorectal condition	74	64.3
	Patients with two anorectal conditions	25	21.7
	Patients with three anorectal conditions	13	11.3
	Patients with ≥4 anorectal conditions	3	2.6
Frequency of Specific Anorectal Conditions *			
	Hemorrhoids	51	29.8
	Obstructive defecation syndrome	37	21.8
	Fecal incontinence	31	18.1
	Rectal prolapse	17	9.9
	Anal fissures	17	9.9
	Anal spasm	10	5.8
	Anal fistula	3	1.8
	Others †	5	2.9

* Total exceeds 115 due to the co-occurrence of multiple conditions. † Includes benign colonic mass, proctitis, colonic polyp, peritoneocele, and diverticulitis.

**Table 3:** Demographic and Clinical Characteristics Associated with Anorectal Disorder in Patients with Urinary Incontinence (*N* = 494).

		With ARD		Without ARD		
Variables		*N*	%	*N*	%	*p*
Total		115	23.3	379	76.7	
Age						0.007 ^a^
	18–29 years	5	16.7	25	83.3	
	30–39 years	15	29.4	36	70.6	
	40–49 years	35	33.0	71	67.0	
	50–64 years	44	23.5	143	76.5	
	≥65 years	16	13.3	104	86.7	
Gender						0.344 ^a^
	Male	7	16.7	35	83.3	
	Female	108	39.9	344	76.2	
Marital Status						0.899 ^a^
	Ever married	103	23.2	341	76.8	
	Non-married	12	24.0	38	76.0	
Smoking Status						0.482 *
	Non-smokers	111	23.5	362	76.5	
	Former smokers	0	0.0	6	100	
	Current smokers	4	26.7	11	73.3	
Weight Status based on BMI						0.877 ^a^
	Underweight/normal weight	19	21.6%	69	78.4%	
	Overweight	30	22.7%	102	77.3%	
	Obese	66	24.1%	208	75.9%	
Having Diabetes						0.844 ^a^
	Yes	38	22.8	129	77.2	
	No	77	23.5	250	76.5	
Having Hypertension						0.014 ^a^
	Yes	28	16.8	139	83.2	
	No	87	26.6	240	73.4	
Having Asthma						0.886 ^a^
	Yes	17	23.9	54	76.1	
	No	98	23.2	325	76.8	
Having Hyperthyroidism						1.00
	Yes	1	20.0	4	80.0	
	No	114	23.3	76.7	379	
Having Hypothyroidism						0.037 ^a^
	Yes	28	31.8	60	68.2	
	No	87	21.4	319	78.6	
Having Dyslipidemia						0.058 ^a^
	Yes	36	18.8	156	81.3	
	No	79	26.2	223	73.8	
Having Any Cardiovascular Disease (CVDs)						0.648 ^a^
	Yes	9	26.5	25	73.5	
	No	106	23.0	354	77.0	
Having Any Neurological Disease						0.657 ^a^
	Yes	8	26.7	22	73.3	
	No	107	23.1	357	76.9	
Having Any Mental or Psychological Condition						0.003 ^a^
	Yes	26	37.1	44	62.9	
	No	89	21.0	335	79.0	
Chronic Constipation						<0.001 ^a^
	Yes	85	39.7	129	60.3	
	No	30	10.7	250	89.3	
Recurrent UTIs						0.316 ^a^
	Yes	31	26.7	85	73.3	
	No	84	22.2	294	77.8	
Previous Pelvic Surgery						0.003 ^a^
	Yes	38	32.8	24	16.8	
	No	78	67.2	119	83.2	
Total Number of Deliveries						0.148 ^a^
	0	19	17.6	104	30.2	
	1–2	10	9.3	32	9.3	
	3–4	31	28.7	69	20.1	
	5–6	27	25.0	75	21.8	
	7–9	16	14.8	46	13.4	
	≥10	5	4.6	18	5.2	
Presence of Anal Sphincter Tear						0.005 *
	Yes	4	3.7	0	0.0	
	No	78	72.2	223	64.8	
	Unknown	26	24.1	121	35.2	
Sexual Dysfunction						<0.001 ^a^
	Yes	22	64.7	12	35.3	
	No	78	19.0	333	81.0	

ARD = Anorectal dysfunction; ^a^ = Chi-square test; * = Fisher exact test.

**Table 4:** Multivariable Logistic Regression Analysis of Anorectal Disorder Predictors.

	Adjusted Odds Ratio (aOR)	95.0% CI		*p*-Value
		Lower Bound	Upper Bound	
Sociodemographic Predictors				
Age	0.977	0.959	0.995	0.014
BMI (kg/m^2^)	1.005	0.969	1.042	0.796
Gender (Male)	0.735	0.307	1.759	0.489
Marital Status (Married)	1.457	0.637	3.334	0.373
Incontinence Type and Pelvic Organ Prolapse Predictors				
Type of UI (Mixed)	0.722	Ref	Ref	0.313
Type of UI (Urge)	1.084	0.625	1.881	0.774
Type of UI (Stress)	1.295	0.708	2.368	0.402
Type of UI (Overflow)	2.143	0.393	11.689	0.379
Cystocele	0.963	0.481	1.926	0.915
Rectocele	6.540	3.179	13.454	<0.001
Clinical and Obstetric Characteristics				
Menstrual status	0.951	0.203	4.450	0.949
Number of deliveries	1.124	0.815	1.550	0.476
Largest infant weight (g)	1.001	1.000	1.003	0.056
Number of abortions	0.846	0.532	1.345	0.480
Previous pelvic surgery	2.265	1.165	4.406	0.016

Dependent outcome variable = presence of any anorectal disorder.

## Data Availability

Data from this study is available for sharing on request.

## Supplementary Materials

The following supporting information can be downloaded at: https://www.mdpi.com/article/10.3390/jcm15031131/s1, Table S1: Clinical, Obstetric, and Pelvic Floor Characteristics Among Urinary Incontinent Patients with and without Anorectal Disorder (ARD).
