# Supplementary material for: Prevalence and Determinants of Anorectal Disorders in Adult Patients with Urinary Incontinence: A Retrospective Cohort Study
Source: J Clin Med. 2026 Feb 1;15(3):1131. doi: 10.3390/jcm15031131 (PMC12898705; doi:10.3390/jcm15031131)
Supplement: Supplementary file 1 [file jcm-15-01131-s001.zip › jcm-4044527-supplementary.pdf]

Table S1. Clinical, Obstetric, and Pelvic Floor Characteristics Among Urinary Incontinent Patients with and without Anorectal Disorder (ARD)

**Variables**	**n (%)**			**P-value**
	**Overall** N = 494	**With ARD** N = 115	**Without ARD** N = 379	
**Type of Urinary Incontinence N= 494**				0.137*
Urge	208 (42.1)	39 (33.9)	169 (44.6)	
Mixed	174 (35.2)	42 (36.5)	132 (34.8)	
Stress	99 (20.0)	29 (25.2)	70 (18.5)	
Overflow	13 (2.6)	5 (4.3)	8 (2.1)	
Pelvic Floor and Obstetric Characteristics (N = 452, women only)				
**Pelvic Organ Prolapse: Cystocele**	452	108	344	<0.001a
Grade 0	78 (17.3)	23 (21.3)	55 (16.0)	
Grade 1	53 (11.7)	22 (20.4)	31 (9.0)	
Grade 2	37 (8.2)	14 (13.0)	23 (6.7)	
Grade 3	27 (6.0)	10 (9.3)	17 (4.9)	
Unknown	257 (56.9)	39 (36.1)	218 (63.4)	
**Pelvic Organ Prolapse: Rectocele**				<0.001*
Grade 0	118 (26.1)	27 (23.1)	91 (26.5)	
Grade 1	34 (7.5)	18 (16.7)	16 (4.7)	
Grade 2	29 (6.4)	15 (13.9)	14 (4.1)	
Grade 3	15 (3.3)	11 (10.2)	4 (1.2)	
Unknown	256 (56.6)	37 (36.1)	219 (63.7)	
**Menstrual Status**				0.011a
Menopause	229 (50.7)	43 (39.8)	186 (54.1)	
Premenopausal	223 (49.3)	65 (60.2)	158 (45.9)	
**Parity**				0.014a
Parous	350 (77.4)	91 (84.3)	259 (75.3)	
Nulliparous	54 (11.9)	13 (12.0)	41 (11.6)	
Unknown	48 (10.6)	4 (3.7)	44 (13.1)	
**Total Number of Deliveries**				
Mean ± SD	3.81 ± 3.24	4.19 ± 3.03	3.68 ± 3.30	0.153b
0	123 (27.2)	19 (17.6)	104 (30.2)	0.147a
1-2	42 (9.3)	10 (9.3)	32 (9.3)	
3-4	100 (22.1)	31 (28.7)	69 (20.1)	
5-6	102 (22.6)	27 (25.0)	75 (21.8)	
7-9	62 (13.7)	16 (14.8)	46 (13.4)	
≥10	23 (5.1)	5 (4.6)	18 (5.2)	
**Delivery: Vaginal Deliveries**				0.100a
0	142 (31.4)	23 (21.3)	119 (34.6)	
1-2	45 (10.0)	12 (11.1)	33 (9.6)	

3-4	102 (22.6)	33 (30.6)	69 (20.1)	
5-6	86 (19.0)	20 (18.5)	66 (19.2)	
7-9	55 (12.2)	15 (13.9)	40 (11.6)	
≥10	22 (4.9)	5 (4.6)	17 (4.9)	
**Delivery: Caesarean Sections**				0.724*
0	367 (81.2)	85 (78.7)	282 (82.0)	
1-2 deliveries	66 (14.6)	18 (16.7)	48 (14.0)	
3-5 deliveries	19 (4.2)	5 (4.6)	14 (4.1)	
**Weight of Largest Infant n=54**				0.210b
Mean ± SD	3444.65 ± 507.60 g	3535.04 ± 576.87	3360.71 ± 427.17	
**Abortions**				<0.001a
0	332 (73.6)	63 (58.3)	269 (78.4)	
1-2	85 (18.8)	34 (31.5)	51 (14.9)	
≥3	34 (7.5)	11 (10.2)	23 (6.7)	
**Previous Pelvic Surgery**				<0.001a
Yes	51 (11.3)	29 (26.9)	22 (6.4)	
No	164 (36.3)	65 (60.2)	99 (28.8)	
Unknown	237 (52.4)	14 (13.0)	223 (64.8)	
**Presence of Anal Sphincter Tear**				0.001*
Yes	4 (0.9)	4 (3.7)	0 (0.0)	
No	301 (66.6)	78 (72.2)	223 (64.8)	
Unknown	147 (32.5)	26 (24.1)	121 (35.2)	
**Sexual Dysfunction**				<0.001a
Yes	28 (6.2)	20 (18.5)	8 (2.3)	
No	376 (83.2)	73 (67.6)	303 (88.1)	
Unknown	48 (10.6)	15 (13.9)	33 (9.6)	

a: Chi-square test; b: Independent samples t-test; *: Fisher's exact test
